# Hepatic Hemangiomas Alter Morphometry and Impair Hemodynamics of the Abdominal Aorta and Primary Branches From Computer Simulations

**DOI:** 10.3389/fphys.2018.00334

**Published:** 2018-04-05

**Authors:** Xiaoping Yin, Xu Huang, Qiao Li, Li Li, Pei Niu, Minglu Cao, Fei Guo, Xuechao Li, Wenchang Tan, Yunlong Huo

**Affiliations:** ^1^Department of Radiology, Affiliated Hospital of Hebei University, Hebei University, Baoding, China; ^2^Department of Mechanics and Engineering Science, College of Engineering, Peking University, Beijing, China; ^3^College of Medicine, Hebei University, Baoding, China; ^4^PKU-HKUST Shenzhen-Hongkong Institution, Shenzhen, China; ^5^Shenzhen Graduate School, Peking University, Shenzhen, China

**Keywords:** hepatic hemangioma, computer tomography, abdominal arterial tree, hemodynamics, morphology

## Abstract

**Background:** The formation of hepatic hemangiomas (HH) is associated with VEGF and IL-7 that alter conduit arteries and small arterioles. To our knowledge, there are no studies to investigate the effects of HH on the hemodynamics in conduit arteries. The aim of the study is to perform morphometric and hemodynamic analysis in abdominal conduit arteries and bifurcations of HH patients and controls.

**Methods:** Based on morphometry reconstructed from CT images, geometrical models were meshed with prismatic elements for the near wall region and tetrahedral and hexahedral elements for the core region. Simulations were performed for computation of the non-Newtonian blood flow using the Carreau-Yasuda model, based on which multiple hemodynamic parameters were determined.

**Results:** There was an increase of the lumen size, diameter ratio, and curvature in the abdominal arterial tree of HH patients as compared with controls. This significantly increased the surface area ratio of low time-averaged wall shear stress (i.e., SAR-TAWSS $=\frac{\text{Surface }{\text{area}}_{\text{TAWSS}\leq 4\text{ dynes}\cdot {\text{cm}}^{-2}}}{\text{Total surface area}}\times$ 100%) (24.1 ± 7.9 vs. 5 ± 6%, 11.6 ± 12.8 vs. < 0.1%, and 44.5 ± 9.2 vs. 21 ± 24% at hepatic bifurcations, common hepatic arteries, and abdominal aortas, respectively, between HH and control patients).

**Conclusions:** Morphometric changes caused by HH significantly deteriorated the hemodynamic environment in abdominal conduit arteries and bifurcations, which could be an important risk factor for the incidence and progression of vascular diseases.

## Introduction

Hemangioma is the most common benign tumor that affects the liver (Gandolfi et al., 1991; John et al., 1994). Hepatic hemangiomas (HH) occur generally in small regions from a few mm to 4 cm despite large HH in the range of 4~40 cm (Sieg et al., 1982; Hoekstra et al., 2013; Toro et al., 2014; Zhao et al., 2015). It was found that HH are associated with microvascular malformations and congenital origin growing slowly from birth (Saegusa et al., 1995; Lehmann et al., 1999). Pregnancy, oral contraceptive use, androgen, or steroid administration were believed to stimulate the incidence and progression of HH (Hoekstra et al., 2013; Toro et al., 2014). The use of specific antibodies against vascular endothelial growth factor (VEGF) and IL-7 was proposed to inhibit the growth of HH (Trastek et al., 1983; Mahajan et al., 2008; Wang et al., 2012). However, the etiology and pathogenesis of HH remain unknown.

Morphometric alteration of large conduit arteries could induce abnormal flow patterns, e.g., stagnation flow, reversal flow, flow vortex, and so on, which were characterized by various hemodynamic risk factors including low time-averaged wall shear stress over a cardiac cycle (low TAWSS), high oscillatory shear index (high OSI), and high TAWSS gradient (high TAWSSG) (Ku, 1997; Kleinstreuer et al., 2001; Huo et al., 2007b, 2008, 2009). Computational fluid dynamics (CFD) has been applied to the hemodynamic analysis in the abdominal arterial tree (Taylor and Draney, 2004). These simulations mainly focused on the flow distribution near stenoses or aneurysms (Boutsianis et al., 2009; Biasetti et al., 2011; Polanczyk et al., 2015; Arzani and Shadden, 2016). To our knowledge, there is lack of studies to show the effects of microvascular malformations on morphometry and hemodynamics in the abdominal aorta and primary branches of HH patients, which could induce hemodynamic impairments to large arteries in the cardiovascular system.

The objective of the study is to demonstrate a comparison of morphometry and hemodynamics in the abdominal aorta and primary branches between HH patients and healthy controls. We hypothesize that HH-induced morphometric changes significantly worsen the hemodynamic environment in abdominal conduit arteries and bifurcations. To test the hypothesis, we reconstructed the large abdominal arterial trees from CT images of 12 HH patients and 9 controls. Flow simulations were performed to solve the Continuity and Navier–Stokes equations with the Carreau-Yasuda model for non-Newtonian blood, similar to a previous study (Yin et al., 2016). The flow velocity waveform was measured at the thoracic aorta of a representative patient and was applied to the inlet of the large abdominal arterial tree with the Womersley velocity profile (Zheng et al., 1985). The fully-developed flow boundary condition was applied to each outlet that considered fractional flow rate (Huo et al., 2013). Based on the computed flow field, multiple hemodynamic parameters, i.e., TAWSS, OSI, and TAWSSG, were determined in the large abdominal arterial trees. Malek et al. showed a threshold TAWSS ≤ 4 dynes/cm^2^; Nordgaard et al. indicated a threshold OSI ≥ 0.15; and Fan et al. proposed a threshold TAWSSG ≥ 500 dynes/cm^3^ for the incidence and progression of atherosclerosis (Malek et al., 1999; Nordgaard et al., 2010; Fan et al., 2016). Hence, we used the surface area ratios of low TAWSS (SAR-TAWSS), high OSI (SAR-OSI), and TAWSSG (SAR-TAWSSG) (see the detailed definitions in the Appendix) to characterize the complex hemodynamic environment in abdominal conduit arteries and bifurcations similar to previous studies (Fan et al., 2016, 2017; Yin et al., 2016).

## Materials and methods

### Study design

**Figure 1:**
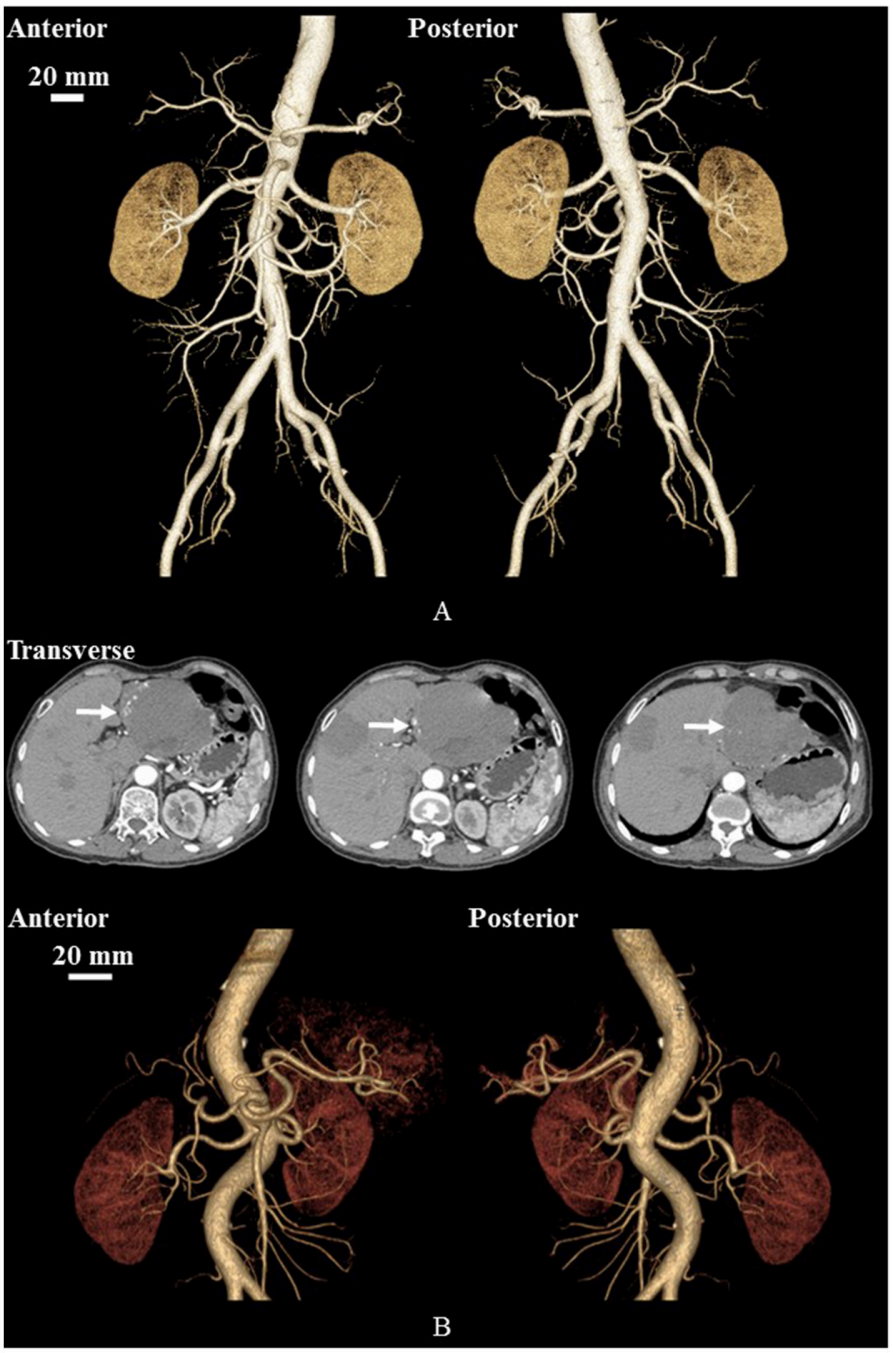
Geometrical models reconstructed from CT images of a representative control **(A)** and a representative HH patient **(B)**. **(B)** Also shows CT-axial angiographs, where arrows mark liver hemangiomas.

We retrospectively analyzed morphometry and hemodynamics of large abdominal arterial trees of HH patients as well as age-matched control subjects, who underwent CT exams at the Affiliated Hospital of Hebei University, China, from January to December 2015. The CT diagnosis of HH in a previous study (Caseiro-Alves et al., 2007) was used, which showed peripheral nodular enhancement at the lesion progressing from the periphery to the center, as shown in Figure 1. A total of 12 HH patients (5 males and 7 females from 28 to 69 years) were compared with a total of 9 controls (7 males and 2 females from 31 to 62 years).

The study was approved by the Institutional Review Board (IRB) for the Affiliated Hospital of Hebei University, which conforms the declaration of Helsinki. All patients gave the signed informed consent for all methods of the study performed in accordance with the relevant guidelines and regulations of the IRB for the Affiliated Hospital of Hebei University.

### Imaging acquisition

CT scans were performed on a Discovery CT750 HD scanner (HDCT, GE Healthcare, Milwaukee, WI, USA). All patients underwent abdomen contrast-enhanced spectral CT from the diaphragm to the edge of the kidney. The spectral CT scan protocol included helical pitch of 0.984:1, rotation speed of 0.5 s, and 50 cm Display Field of View (DFOV). The nonionic contrast media (ioversol, 320 mgl/ml) at the dose of 1.0 ml/kg was injected with a power injector at a rate of 3.0 ml/s through median cubital vein. Arterial phase scanning with an automatic tracking technique with automated scan-triggering software (SmartPrep, GE Healthcare, Milwaukee, WI, USA) started 8 s after the trigger threshold (100 HU) was reached at the level of the celiac trunk (He et al., 2014). The portal venous phase scanning and later delay phase scanning started for 30 and 120 s, respectively, after the beginning of the arterial phase scanning.

### Geometrical model

**Figure 2:**
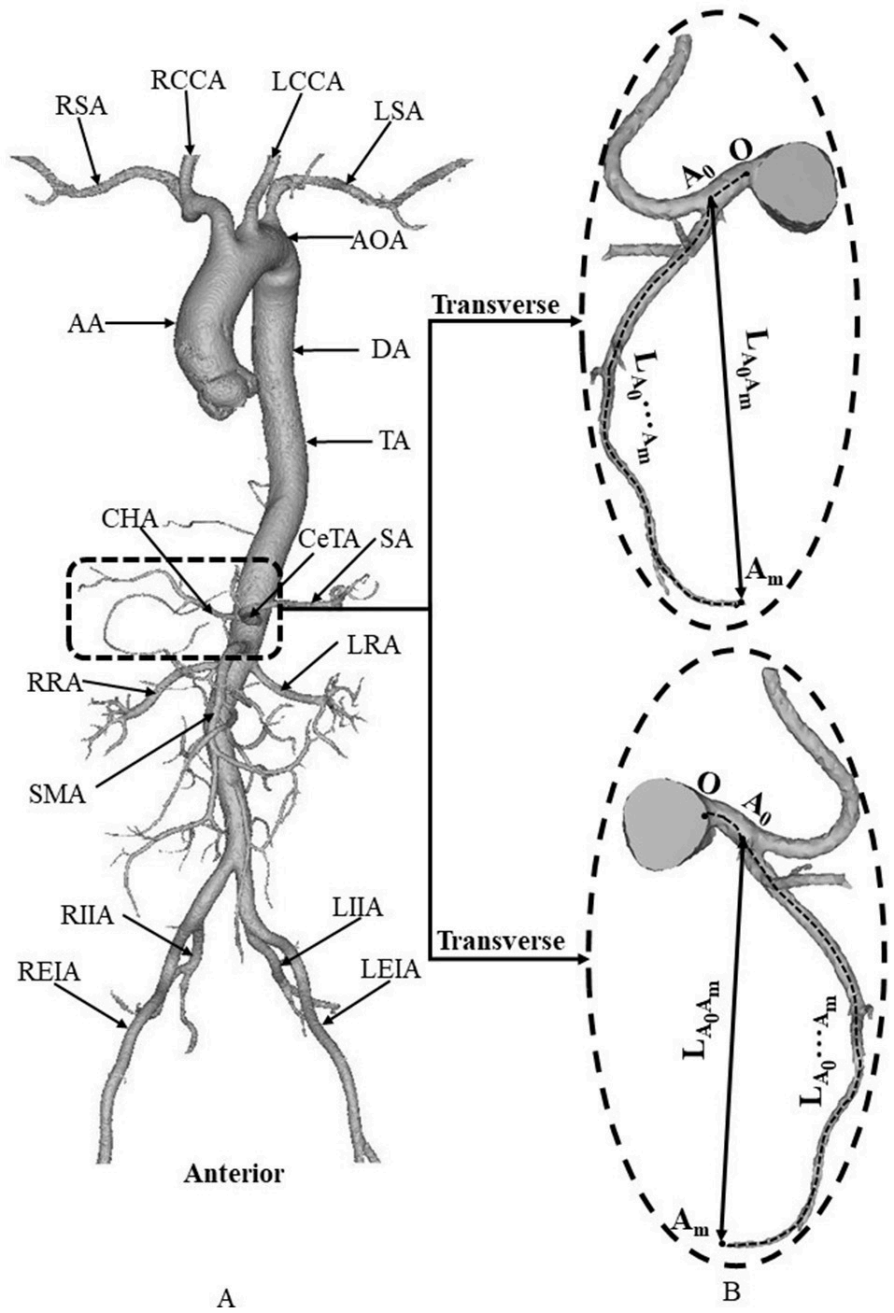
**(A)** Schematic diagram of aorta and primary branches, where AA, ascending aorta; AOA, arch of aorta; DA, descending aorta; TA, thoracic aorta; RSA, right subclavian artery; RCCA, right common carotid artery; LCCA, left common carotid artery; LSA, left subclavian artery; CeTA, celiac trunk artery; CHA, common hepatic artery; SA, splenic artery; RRA, right renal artery; LRA, left renal artery; SMA, superior mesenteric artery; RIIA, right internal iliac artery; REIA, right external iliac artery; LIIA, left internal iliac artery; LEIA, left external iliac artery; and **(B)** zoomed diagram of CeTA and CHA, where the black dotted line represents the centerline, *A*_0_ refers to the intersection of centerlines between CeTA and CHA, and *L*_*A*_0_*A*_*m*__ and *L*_*A*_0_…*A*_*m*__denote the linear and arc lengths from point *A*_0_ to *A*_*m*_, respectively.

Morphometric data of the abdominal aorta and primary branches were extracted from patient CT images using the MIMICS software (Materialise, NV, Belgium), as shown in Figure 2. A centerline was formed by a series of center points which were located in the center on the cross–sectional views of the contour of the 3D vessel. Subsequently, the best fit diameter, D_fit_, was calculated as twice the average radius between the point on the centerline and the contour forming the contour of the 3D vessel (Fan et al., 2016; Huang et al., 2016). The CT reconstruction and imaging analysis were performed by researchers (X. Huang, Q. Li and M. Cao) at the College of Engineering, Peking University and (F. Guo and X. Li) at the College of Medicine, Hebei University as well as a Radiologist (X. Yin) at the Affiliated Hospital of Hebei University. The reproducibility of the measurements showed κ value equals to 0.87.

### Mesh generation

Based on morphometric data, geometrical models were meshed with prismatic elements for the near wall region (number of layers = 3, height ratio = 1.2, total height = 1 mm) and tetrahedral and hexahedral elements for the core region (maximal element size = 0.3 mm) using the ANSYS ICEM software (ANSYS Inc., Canonsburg, USA) and then smoothed using the Geomagic Studio software (3D Systems, Rock Hill, USA). Mesh dependency and skewness and orthogonal quality metrics were analyzed to satisfy the quality of the grids such that the relative error in two consecutive mesh refinements was < 1% for the maximum WSS and velocity of steady state flow with inlet flow velocity equal to the time-averaged velocity over a cardiac cycle, similar to previous studies (Chen et al., 2016; Fan et al., 2016; Yin et al., 2016). A total of 3.5–4.5 million hybrid volume elements were necessary to accurately mesh the computational domain. Simulations were demonstrated in a workstation with 3GHz dual Xeon processor and 32-GB of memory, and the computational time for each model ranged from 48 to 72 h.

### Computational model

Similar to a previous study (Biasetti et al., 2011), the Carreau-Yasuda model for the non-Newtonian blood flow was performed as:

(1) $$\frac{\mu -\mu_{\infty }}{\mu_{0}-\mu_{\infty }}={\left[1+{\left(\lambda \dot{\gamma }\right)}^{a}\right]}^{\left(n-1\right)/a}$$

where μ_0_ and μ_∞_ equal to 0.16 and 0.0035 Pa·s while λ (8.2 s), *n* (0.02128), and *a* (0.64) refer to the relaxation time, power-law index and Yasuda exponent, respectively. The viscosity (μ) is associated with the shear rate ($\dot{\gamma }$). The flow velocity waveform was measured at the thoracic aorta of a female patient (Table 1. HH Group, No.6) through MRI scans with a one-component cine phase-contrast sequence in a 1.5 T scanner (GE Signa SP, GE Medical Systems, Milwaukee, WI). The velocity waveform was scaled to the Womersley profile (i.e., Equation 3 in Zheng et al., 1985) as the inlet boundary condition. The fully-developed flow boundary condition was set to each outlet, where the diffusion flux for all flow variables in the exit direction are zero. Considering the high number of outflow branches, the flow rate weighting factor (i.e., the sum of flow rate weighting factors is 100%) was set to each outlet. The flow rate weighting factor was set to 24% at the distal abdominal aorta (Xiao et al., 2013) while the weighting factors at other outlets were determined by the flow-diameter scaling law (i.e., *Q* ∝ *D*^7/3^) (Huo and Kassab, 2012a, 2016; Fan et al., 2016).

**Table 1:** Demographics and morphometry in each patient of control and HH groups.

**Groups**	**No**	**Sex**	**Age**	***D_CHA_* (mm)**	***D_CeTA_* (mm)**	***D_TA_* (mm)**	***L*_*A*_0_*A*_*m*__ (mm)**	***L*_*A*_0_⋯*A*_*m*__ (mm)**	$\frac{D_{CHA}}{D_{CeTA}}$	$\frac{D_{CHA}}{D_{TA}}$	$\frac{D_{CeTA}}{D_{TA}}$	$\frac{L_{A_{0}A_{m}}}{L_{A_{0}\cdots A_{m}}}$
Control group	1	M	31	3.14	5.47	18.11	17.17	17.79	0.57	0.17	0.3	0.96
	2	M	37	3.02	7.16	20.23	58.39	84.53	0.42	0.15	0.35	0.69
	3	M	40	2.62	5.08	17.83	25.96	37.91	0.52	0.15	0.28	0.68
	4	M	46	2.96	5.77	20.64	41.47	53.21	0.51	0.14	0.28	0.78
	5	M	52	3.32	7.04	18.76	50.62	53.74	0.47	0.18	0.38	0.94
	6	M	52	1.99	5.93	18.14	46.43	60.47	0.34	0.11	0.33	0.77
	7	F	55	2.9	4.86	19.85	35.73	41.55	0.6	0.15	0.24	0.86
	8	M	58	3.34	4.59	20.02	33.43	33.84	0.73	0.17	0.23	0.99
	9	F	62	2.91	6.35	23.78	32.78	39.03	0.46	0.12	0.27	0.84
HH group	1	F	28	7.85	6.58	17.76	46.85	55.29	1.19	0.44	0.37	0.85
	2	M	42	4.41	7.07	21.49	46.52	75.68	0.62	0.21	0.33	0.61
	3	M	43	4.88	7.54	23.76	59.23	94.12	0.65	0.21	0.32	0.63
	4	F	50	3.85	6.94	17.54	33.43	40.67	0.55	0.22	0.4	0.82
	5	M	50	4.81	7.77	21.67	52.97	66.36	0.62	0.22	0.36	0.80
	6	F	50	4.21	5.94	23.3	71.82	97.12	0.71	0.18	0.25	0.74
	7	F	52	3.65	4.76	21.34	32.08	51.73	0.77	0.17	0.22	0.62
	8	F	62	5.51	5.94	19.34	36.22	44.57	0.93	0.28	0.31	0.81
	9	M	63	4.92	7.24	19.97	25.07	45.48	0.68	0.25	0.36	0.55
	10	F	65	2.46	4.52	21.75	29.06	48.06	0.54	0.11	0.21	0.60
	11	M	67	5.84	7.61	25.75	37.81	54.46	0.77	0.23	0.3	0.69
	12	F	69	3.76	7.08	23.69	40.95	69.94	0.53	0.16	0.3	0.59

The mean Reynolds number (averaged over a cardiac cycle) at the inlet of thoracic aorta has values of 1028 ± 101 and 1065 ± 163 in control and HH groups, respectively. The commercial software solver FLUENT (ANSYS, Inc., Canonsburg, USA) was used to solve the Navier-Stokes and continuity equations as:

(2) $$\nabla \cdot \vec{u}=0$$

(3) $$\rho \frac{\partial \vec{u}}{\partial t}+\rho \vec{u}\cdot \nabla \vec{u}=-\nabla p+\mu \nabla^{2}\vec{u}$$

where *p*, $\vec{u}$, and ρ (= 1,060 kg/m^3^) represent the pressure, velocity, and blood density, respectively. An implicit second-order backward Euler method was used with a time step 0.01 s. The convergence of solutions was guaranteed by the globe balance of the conservation equations with a RMS (Root Mean Squared) residual criterion of 10^−3^. Four cardiac cycles (0.84 s per cardiac cycle) were conducted to achieve the convergence for the transient analysis similar to previous studies (Fan et al., 2016). Hemodynamic parameters including TAWSS, OSI, and TAWSSG were determined from the computed flow fields in the fourth cardiac cycle, consistent with previous studies (Chen et al., 2016; Fan et al., 2016; Yin et al., 2016). Moreover, we computed SAR-TAWSS, SAR-OSI, and SAR-TAWSSG in abdominal conduit arteries and bifurcations.

### Statistical analysis

The mean ± *SD* (standard deviation) values of various morphometric and hemodynamic parameters were computed by averaging over all subjects in a population. A two-way ANOVA (SigmaStat 3.5) was used to detect the statistical difference of the parameters between control and HH groups. A *p*-value < 0.05 was indicative of a significant difference between two populations.

## Results

Figure 2 shows a schematic representative of aorta and primary branches. Table 1 lists the morphometry in each patient of control and HH groups, which have mean ± SD ages of 48 ± 10 and 54 ± 14 years, respectively. As shown in Table 2, parameters, *D*_*CHA*_, *D*_*CeTA*_ and *D*_*TA*_ refer to the diameters averaged along the entire length of common hepatic artery, celiac trunk artery, and thoracic aorta, respectively. They have mean ± *SD* values of 2.9 ± 0.4, 5.8 ± 0.9, and 19.7 ± 1.9 mm in the control group and 4.7 ± 1.4, 6.6 ± 1.1, and 21.5 ± 2.5 in the HH group, which show significant difference of *D*_*CHA*_ (*p*-value < 0.05). The diameters of CeTA and TA in HH patients are higher than those in controls despite no statistical difference. Moreover, the diameter ratios, $\frac{D_{CHA}}{D_{CeTA}}$ and $\frac{D_{CHA}}{D_{TA}}$, in the HH group are significantly higher than the control group (*p*-value < 0.05). On the other hand, there are significantly lower ratios of linear to arc lengths ($\frac{L_{A_{0}A_{m}}}{L_{A_{0}{\cdots A}_{m}}}$) in HH CHAs compared with controls (0.69 ± 0.11 vs. 0.84 ± 0.11, *p*-value < 0.05). This indicates severe curvatures in CHAs of HH patients.

**Table 2:** Statistical analysis of morphometric and hemodynamic parameters in control and HH groups.

**Groups**		**Control group**	**HH group**	***p*-value**
**MORPHOMETRIC ANALYSIS**				
*D*_*CHA*_ (mm)	Mean ± SD	2.9 ± 0.4	4.7 ± 1.4	<0.05
	95% CI	2.6–3.2	3.8–5.5	
*D*_*CeTA*_ (mm)	Mean ± SD	5.8 ± 0.9	6.6 ± 1.1	0.099
	95% CI	5.1–6.5	5.9–7.3	
*D*_*TA*_ (mm)	Mean ± SD	19.7 ± 1.9	21.5 ± 2.5	0.094
	95% CI	18.3–21.1	19.9–23.0	
*L*_*A*_0_*A*_*m*__ (mm)	Mean ± SD	38 ± 12.7	42.7 ± 13.6	0.432
	95% CI	28.2–47.8	34.1–51.3	
*L*_*A*_0_⋯*A*_*m*__ (mm)	Mean ± SD	46.9 ± 19	62.0 ± 19.0	0.088
	95% CI	32.3–61.5	49.9–74.0	
$\frac{D_{CHA}}{D_{CeTA}}$	Mean ± SD	0.51 ± 0.11	0.71 ± 0.19	<0.05
	95% CI	0.43–0.6	0.59–0.83	
$\frac{D_{CHA}}{D_{TA}}$	Mean ± SD	0.15 ± 0.02	0.22 ± 0.08	<0.05
	95% CI	0.13–0.17	0.17–0.27	
$\frac{D_{CeTA}}{D_{TA}}$	Mean ± SD	0.3 ± 0.05	0.31 ± 0.06	0.566
	95% CI	0.26–0.33	0.27–0.35	
$\frac{L_{A_{0}A_{m}}}{L_{A_{0}{\cdots A}_{m}}}$	Mean ± SD	0.84 ± 0.11	0.69 ± 0.11	<0.05
	95% CI	0.75–0.92	0.63–0.76	
**HEMODYNAMIC ANALYSIS**				
D_Inlet_ (mm)		21.5 ± 2.1	22.3 ± 3.4	0.704
Re_mean_		1028 ± 101	1065 ± 163	0.704
**AREA RATIOS AT BIFURCATIONS BETWEEN CeTA AND CHA AND SA**				
SAR-TAWSS (%)		4 ± 5	14.3 ± 4.4	<0.05
SAR-TAWSSG (%)		27 ± 38	36.1 ± 28.5	0.678
**AREA RATIOS IN CHAs**				
SAR-TAWSS (%)		<0.1	6.3 ± 5.1	<0.05
SAR-TAWSSG (%)		68 ± 33	55.4 ± 17.1	0.445
**AREA RATIOS IN ABDOMINAL AORTAS**				
SAR-TAWSS (%)		17 ± 22	43.6 ± 8.4	<0.05
SAR-TAWSSG (%)		1 ± 1	2.1 ± 1.3	0.17

**Figure 3:**
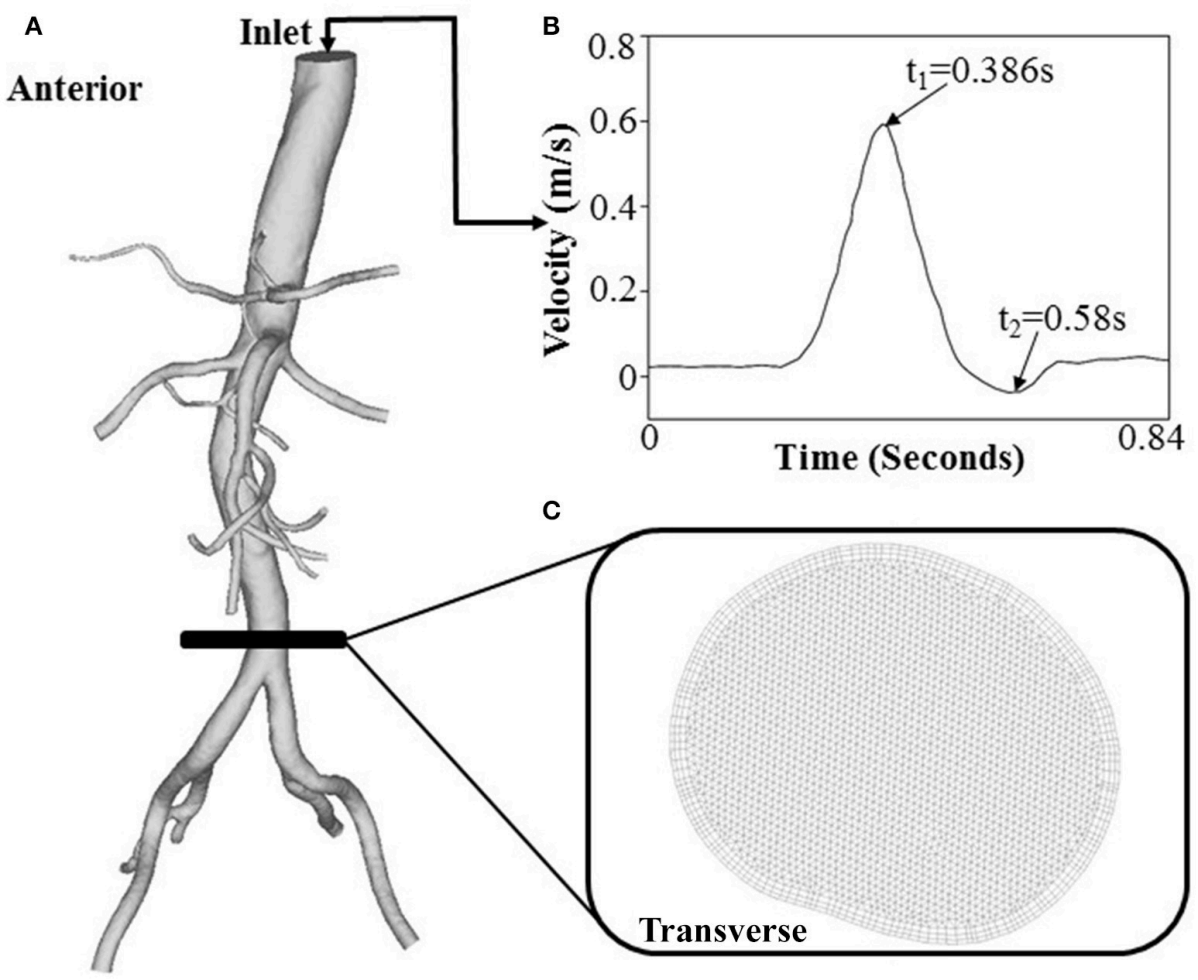
**(A)** A geometrical model for flow simulations, **(B)** the inlet flow waveform measured at a patient TA by phase-contrast MRI, and **(C)** FE meshes at a cross-sectional area.

**Figure 4:**
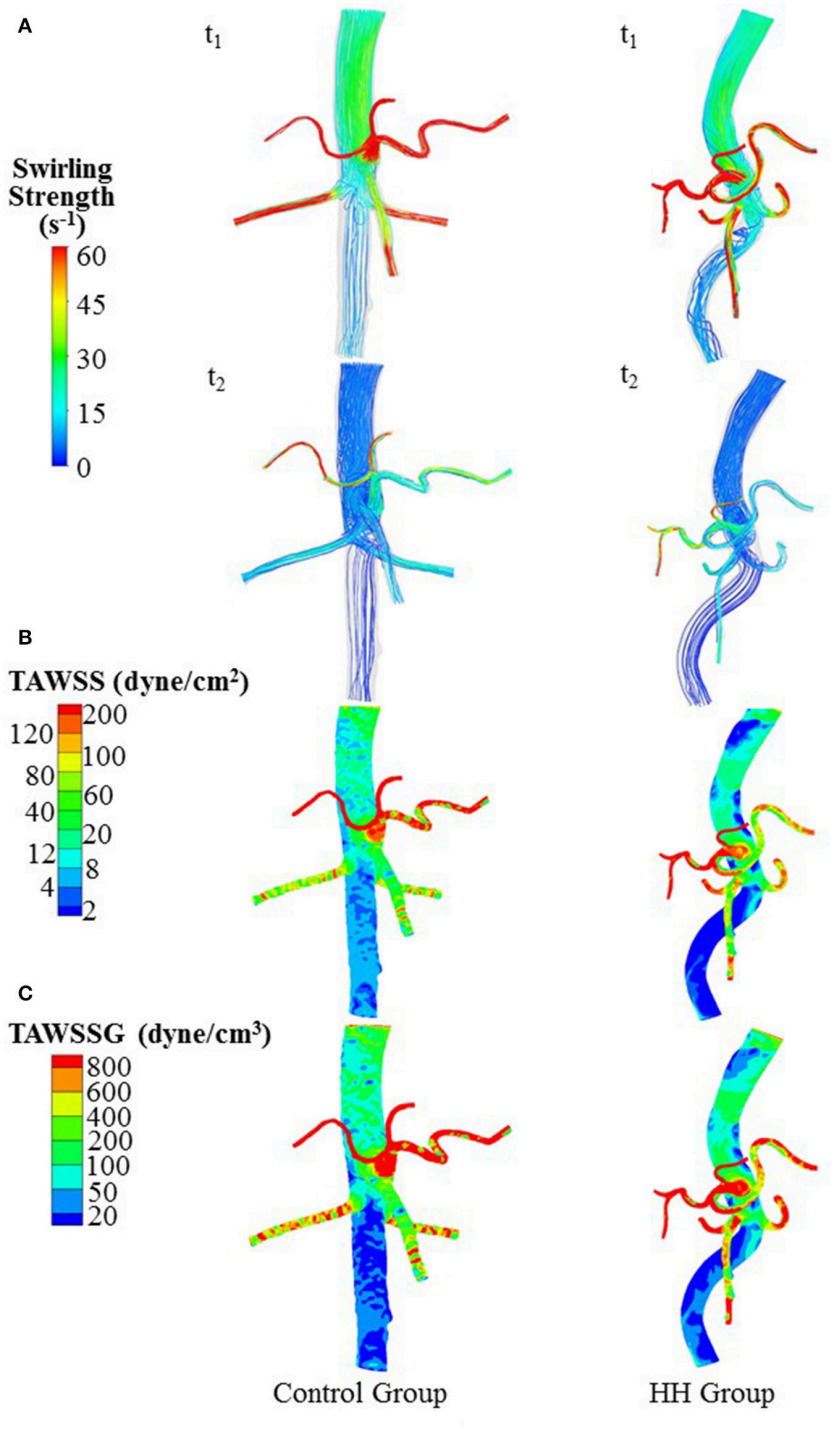
**(A)** Flow streamlines (unit: s^−1^) at time instances with the highest and lowest flow velocities at the inlet of TA (i.e., t_1_ and t_2_ in Figure 3B), **(B)** TAWSS (unit: dynes/cm^2^), and **(C)** TAWSSG (unit: dynes/cm^3^) in a representative control and a representative HH patient.

**Figure 5:**
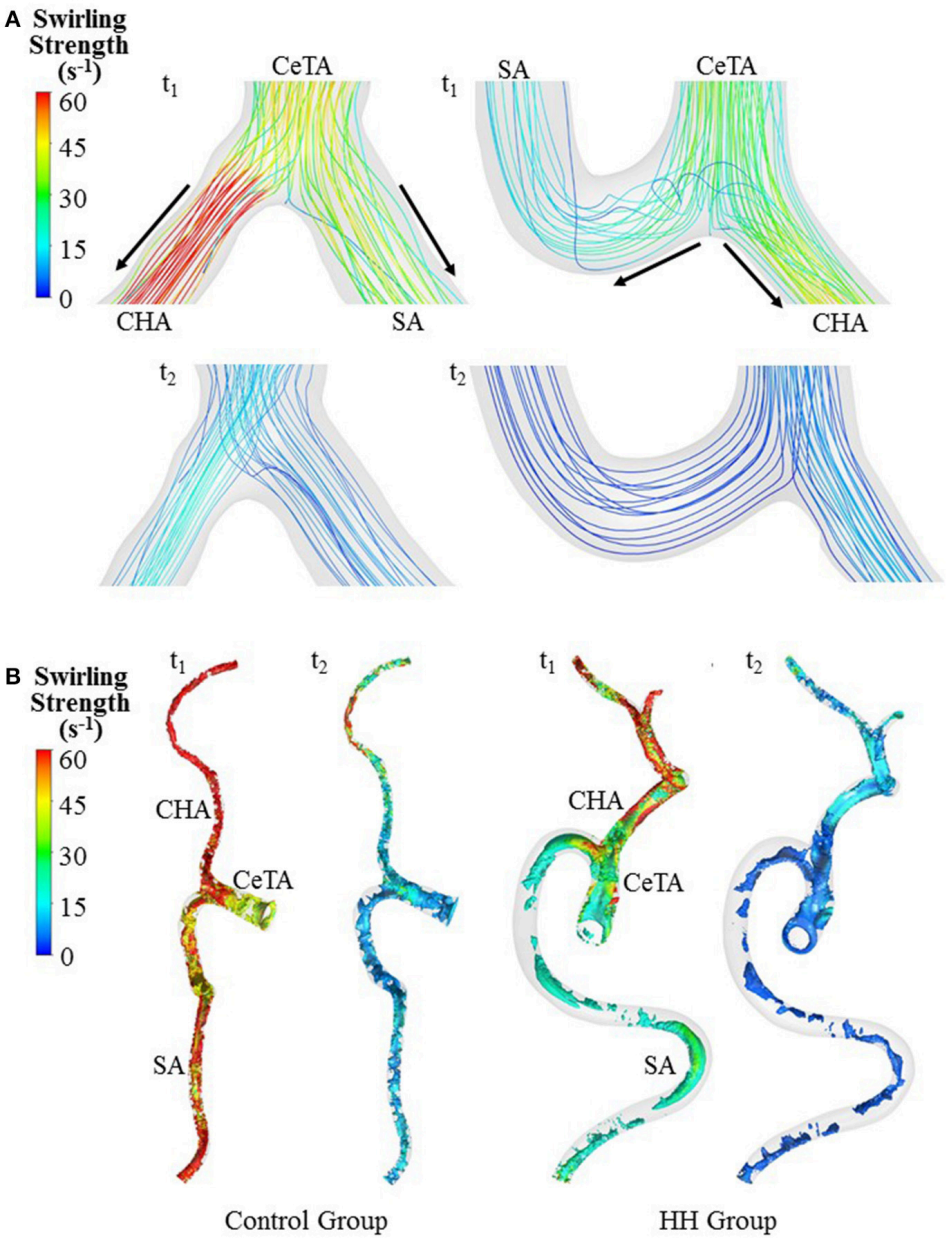
Bifurcation flow streamlines **(A)** and vortex cores **(B)** (unit: s^−1^) in a representative control and a representative HH patient at time instances with the highest and lowest flow velocities at the inlet of TA.

The Reynolds number at the inlet of thoracic aorta with diameters of 21.5 ± 2.1 and 22.3 ± 3.4 mm has values of 1.028 ± 101 and 1.065 ± 163 in control and HH groups, respectively. Hence, the simulation was carried out for computation of flow fields, based on the geometrical models and boundary conditions in Figure 3. The highest and lowest flow velocities at the inlet of TA occur at time instances of t_1_ and t_2_, as shown in Figure 3B. Figures 4A–C show the flow streamlines (unit: s^−1^) at time instances of t_1_ and t_2_, TAWSS (unit: dynes/cm^2^) and TAWSSG (unit: dynes/cm^3^), respectively, in abdominal arterial trees of a representative control as well as a representative HH patient. Accordingly, Figures 5A,B show the flow streamlines and vortex cores (unit: s^−1^) at the bifurcation between the celiac trunk artery and common hepatic and splenic arteries. At some time instances in a cardiac cycle, stagnation flow as well as slight reversal flow occurs in three major sites: opposite to flow divider, lateral to junction orifice, and inner curvature, which results in a low TAWSS. Moreover, transient secondary flow leads to complex flow patterns near the bifurcation carina. Hence, we computed SAR-TAWSS and SAR-TAWSSG to analyze the hemodynamics in CHAs and abdominal aortas and at bifurcations between CeTA and CHA and SA in the two groups, as shown in Table 2. Since SAR-OSI < 1%, it is neglected here. Hepatic hemangiomas significantly increase SAR-TAWSS (14.3 ± 4.4 vs. 4 ± 5 at bifurcations between CeTA and CHA and SA, 6.3 ± 5.1 vs. < 0.1 in CHAs, and 43.6 ± 8.4 vs. 17 ± 22 in abdominal aortas for HH vs. control groups, *p*-value < 0.05) and impair the hemodynamics in the large abdominal arterial tree. There is no statistical difference of SAR-TAWSSG between control and HH groups.

## Discussion

The study compared morphometry and hemodynamics in the abdominal aorta and primary branches between control and HH patients. The major findings are reported as: (1) HH increase the lumen size in the abdominal arterial tree significantly and show higher $\frac{D_{CHA}}{D_{CeTA}}$ and $\frac{D_{CHA}}{D_{TA}}$ in comparison with controls (*p*-value < 0.05); (2) HH lead to severe curvature in CHAs; and (3) HH result in a significant increase of SAR-TAWSS due to abnormal flow patterns in CHAs and abdominal aortas as well as at bifurcations between CeTA and CHA and SA. The findings are discussed in relation to the potential incidence and progression of cardiovascular diseases.

The prevalence of HH ranges from 0.4 to 7.3% of all space-occupying hepatic lesions (Ishak and Rabin, 1975; John et al., 1994). Since benign HH of small size received little attention (John et al., 1994), previous studies emphasized on the treatment of large HH that cause clinical symptoms (Cui et al., 2003; Zagoria et al., 2004; Tak et al., 2006) and neglected potential impairments of small HH. To our knowledge, the present study first investigates the effects of small HH on morphometry and hemodynamics of the large abdominal arterial tree.

The CT reconstruction showed an increase of lumen size and curvature in the abdominal aorta and primary branches of HH patients. There is higher expression of VEGF and IL-7 in HH patients, which contribute to microvascular malfunctions (Trastek et al., 1983; Mahajan et al., 2008; Wang et al., 2012). The overexpression of VEGF and IL-7 can also be critical risk factors for the morphometric changes of large abdominal arterial trees (Lu and Kassab, 2011; Kim and Byzova, 2014). Moreover, HH are comprised of a chaotic enlargement of distorted micro-vessels such that the flow overload in large conduit arteries occurs to satisfy the balance of supply and demand. The remodeling of conduit arteries in flow overload should conform to the “uniform normal and shear stress hypothesis” (Kassab et al., 2002; Huo et al., 2007a; Huo and Kassab, 2012b), which results in the increased lumen size and curvature of abdominal conduit arteries (Garcia and Kassab, 1985).

A key finding is the deteriorated hemodynamic environment at bifurcations between CeTA and CHA and SA and in abdominal aortas and CHAs of HH patients. In comparison with controls, the significantly increased $\frac{D_{CHA}}{D_{CeTA}}$ induced more complex flow patterns (e.g., stagnation flow, reversal flow, and second flow) at bifurcations between CeTA and CHA and SA in HH patients, as shown in Figure 5. Here, SAR-TAWSS, SAR-OSI, and SAR-TAWSSG were used to characterize the complexity of flow patterns similar to previous studies (Chen et al., 2016; Fan et al., 2016; Yin et al., 2016). The 5-fold increase of SAR-TAWSS and unchanged SAR-OSI and SAR-TAWSSG indicated the deteriorated hemodynamics at bifurcations of HH patients. On the other hand, we have shown low TAWSS on the inner curvature mainly due to the stagnation flow (Huo et al., 2007b, 2008, 2009). The HH-induced curvature significantly increased the values of SAR-TAWSS in both abdominal aortas and CHAs. Therefore, HH impaired the hemodynamics in the large abdominal arterial tree.

### Potential implications

Low TAWSS, high OSI, and TAWSSG can stimulate the vessel wall to induce abnormal biochemical signals relevant to multiple cardiovascular diseases (Chiu and Chien, 2011). Since most of HH are small and benign, previous studies focused on the microvascular malfunctions and microcirculation, but neglected the possibilities of hemodynamic impairments to abdominal conduit arteries. The present study implies that HH-induced morphometric and hemodynamic changes could lead to the incidence and progression of various vascular diseases (e.g., intimal thickening, atherosclerosis, vascular stiffness, and so on) in abdominal conduit arteries. Moreover, this study implies that the use of antibodies against VEGF and IL-7 could inhibit the development of HH and vascular diseases occurring in large conduit arteries.

### Critique of the study

The retrospective data were collected from a single center such that the sample size of HH and control patients was relatively small. For further hemodynamic analysis, the large sample size should be collected from multiple centers to ensure the questionnaire sample requirement. Furthermore, prospective studies are required to investigate the effects of HH on various vascular diseases occurring in large conduit arteries. Although the simulation with non-Newtonian model was used to compute the pulsatile blood flow, we neglected the effects of vessel compliance. The FSI (i.e., fluid-structure interaction) simulation should be carried out and validated against the measurements from MRI or 4D flow to investigate hemodynamic changes relevant to the HH-induced abdominal stiffness in the following study.

## Conclusions

The retrospective study showed the changes of morphometry and hemodynamics in HH patients compared with healthy controls. It was found that HH increased lumen size and curvature of abdominal conduit arteries as well as diameter ratios. The altered morphometry significantly deteriorated the hemodynamics in the large abdominal arterial tree, i.e., an increase of SAR-TAWSS due to abnormal flow patterns in CHAs and abdominal aortas and at bifurcations between CeTA and CHA and SA of HH patients. This implied the possibilities of HH-induced hemodynamic impairments to abdominal conduit arteries. These findings can also improve our understanding of hemodynamic mechanisms relevant to the etiology and pathogenesis of HH.

## Author contributions

The CT reconstruction and imaging analysis were performed by XH, QL, LL, PN, and MC at the College of Engineering, Peking University and FG and XL at the College of Medicine, Hebei University as well as a Radiologist XY at the Affiliated Hospital of Hebei University. Paper was drafted and revised by YH and WT.

### Conflict of interest statement

The authors declare that the research was conducted in the absence of any commercial or financial relationships that could be construed as a potential conflict of interest.

## Appendix

1. **SAR-TAWSS at the bifurcation**: surface area ratio of low TAWSS ($\text{=}\frac{\text{Surface }{\text{area}}_{\text{TAWSS}\leq \text{4 dynes}\cdot {\text{cm}}^{-\text{2}}}}{\text{Bifurcation surface area}}\times 100\%$) at the bifurcation between the celiac trunk artery and common hepatic and splenic arteries. The end-to-side “bifurcation surface area” is defined as the surface area of the proximal graft (5 mm length) to the bifurcation center plus the surface area of the distal arteries (5 mm length) to the bifurcation center. Surface area of TAWSS ≤ 4 dynes/cm^2^ refers to the disease-prone site (Malek et al., 1999).
2. **SAR-TAWSS in the artery**: surface area ratio of low TAWSS ($\text{=}\frac{\text{Surface }{\text{area}}_{\text{TAWSS}\leq \text{4 dynes}\cdot {\text{cm}}^{-\text{2}}}}{\text{Surface area of the artery}}\times 100\%$) in the artery. “Surface area of the artery” refers to the surface area of the celiac trunk artery (or abdominal aorta).
3. **SAR-OSI at the bifurcation**: surface area ratio of high OSI($\text{=}\;\frac{\text{Surface }{\text{area}}_{\text{OSI}\geq \text{0}.\text{15}}}{\text{Bifurcation surface area}}\times 100\%$) at the bifurcation between the celiac trunk artery and common hepatic and splenic arteries. Surface area of OSI ≥ 0.15 refers to the disease-prone site (Fan et al., 2016).
4. **SAR-OSI in the artery**: surface area ratio of high OSI ($\text{=}\frac{\text{Surface }{\text{area}}_{\text{OSI}\geq \text{0}.\text{15}}}{S\text{urface area of the artery}}\times 100\%$) in the artery.
5. **SAR-TAWSSG at the bifurcation**: surface area ratio of high TAWSSG ($\text{=}\frac{\text{Surface }{\text{area}}_{\text{TAWSSG}\geq \text{500 dynes}\cdot {\text{cm}}^{-\text{3}}}}{\text{Bifurcation surface area}}\times 100\%$) at the bifurcation between the celiac trunk artery and common hepatic and splenic arteries. Surface area of TAWSSG ≥ 500 dynes/cm^3^ refers to the disease-prone site (Fan et al., 2016).
6. **SAR-TAWSSG in the artery**: surface area ratio of high TAWSSG ($\text{=}\frac{\text{Surface }{\text{area}}_{\text{TAWSSG}\geq \text{500 dynes}\cdot {\text{cm}}^{-\text{3}}}}{\text{Surface area of the artery}}\times 100\%$) in the artery.
